# *N* ‐Acetylcysteine protects against cobalt chloride‐induced endothelial dysfunction by enhancing glucose‐6‐phosphate dehydrogenase activity

**DOI:** 10.1002/2211-5463.13449

**Published:** 2022-06-20

**Authors:** Chen Yang, Xiaofang Zhang, Xilin Ge, Chunmei He, Suhuan Liu, Shuyu Yang, Caoxin Huang

**Affiliations:** ^1^ Department of Endocrinology and Diabetes, Xiamen Diabetes Institute, Fujian Key Laboratory of Translational Research for Diabetes,The First Affiliated Hospital of Xiamen University, School of Medicine Xiamen University Xiamen China; ^2^ Department of Geriatrics, Jinling Hospital Medical School of Nanjing University China; ^3^ Research Center for Translational Medicine The First Affiliated Hospital of Xiamen University China; ^4^ Traditional Chinese Medicine Research Studio The First Affiliated Hospital of Xiamen University China

**Keywords:** endothelial cells, glucose‐6‐phosphate dehydrogenase, hypoxia, *N*‐Acetylcysteine, pentose phosphate pathway

## Abstract

Hypoxia‐induced endothelial dysfunction is known to be involved in the pathogenesis of several vascular diseases. However, it remains unclear whether the pentose phosphate pathway (PPP) is involved in regulating the response of endothelial cells to hypoxia. Here, we established an *in vitro* model by treating EA.hy926 (a hybrid human umbilical vein cell line) with cobalt chloride (CoCl_2_; a chemical mimic that stabilizes HIF‐1α, thereby leading to the development of hypoxia), and used this to investigate the involvement of PPP by examining expression of its key enzyme, glucose‐6‐phosphate dehydrogenase (G6PD). We report that CoCl_2_ induces the accumulation of HIF‐1α, leading to endothelial cell dysfunction characterized by reduced cell viability, proliferation, tube formation, and activation of cytokine production, accompanied with a significant decrease in G6PD expression and activity. The addition of 6‐aminonicotinamide (6‐AN) to inhibit PPP directly causes endothelial dysfunction. Additionally, *N*‐Acetylcysteine (NAC), a precursor of glutathione, was further evaluated for its protective effects; NAC displayed a protective effect against CoCl_2_‐induced cell damage by enhancing G6PD activity, and this was abrogated by 6‐AN. The effects of CoCl_2_ and the involvement of G6PD in endothelial dysfunction have been confirmed in primary human aortic endothelial cells. In summary, G6PD was identified as a novel target of CoCl_2_‐induced damage, which highlighted the involvement of PPP in regulating the response of endothelial cell CoCl_2_. Treatment with NAC may be a potential strategy to treat hypoxia or ischemia, which are widely observed in vascular diseases.

## Graphical abstract

In this study, G6PD was identified as a novel candidate in CoCl_2_‐induced endothelial cell damage, including abrogated cell viability, proliferation, tube formation, and increased inflammatory/angiogenetic cytokines. Pretreatment with NAC can activate G6PD activity and ameliorate CoCl_2_‐induced endothelial cell damage.

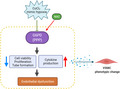

**Abbreviations**
6‐AN: 6‐aminonicotinamide
ALEs: advanced lipoxidation end products
CAT: catalase
CoCl_2_
: cobalt chloride
G6PD: glucose‐6‐phosphate dehydrogenase
GPX1: glutathione peroxidase 1
GSR: glutathione‐disulfide reductase
GSS: glutathione synthetase
HIF‐1: hypoxia‐inducible factor‐1
HO‐1: heme oxygenase 1
I/RI: ischemia reperfusion injury
NAC: *N*‐Acetylcysteine
NQO1: NAD(P)H quinone dehydrogenase 1
PDR: proliferative diabetic retinopathy
PHD: prolyl hydroxylases
PPP: pentose phosphate pathway
SOD1: superoxide dismutase 1
SOD2: superoxide dismutase 2
VHL: von Hippel–Lindau
VSMC: vascular smooth muscle cells

Blood vessels play a fundamental role in the transport of oxygen and nutrients to all tissues in the body. Hypoxia or ischemia is widely observed in vascular issues, such as diabetic vascular complications, obstructive sleep apnea, pulmonary hypertension, and myocardial ischemia reperfusion injury (I/RI) [1, 2, 3]. As the key component of vasculatures, endothelial cells line in the internal lumen of vessels and directly come in contact with vascular smooth muscle cells (VSMCs) or pericytes to form a barrier between circulating blood and tissues and regulate vessel functions. Thus, endothelial cells are directly exposed to oxygen changes in tissues during development processes or diseases, which may disturb cell viability, proliferation, tube‐forming capacity, and barrier integrity. In addition, increasing evidences have revealed that hypoxia can rapidly activate endothelial cells to release inflammatory cytokines, further exacerbating the pathogenesis of hypoxic tissues [4, 5, 6, 7, 8]. Therefore, it is important to identify the common mechanisms of hypoxia‐induced endothelial dysfunction for therapeutic purposes.

The pentose phosphate pathway (PPP) is a metabolic pathway, parallel to glycolysis, that metabolizes glucose into NADPH and ribose 5‐phosphate to maintain redox balance and nucleotide synthesis, respectively [9, 10, 11, 12]. Glucose‐6‐phosphate dehydrogenase (G6PD) is the first rate‐limiting enzyme of PPP that convert glucose‐6‐phosphate into 6‐phosphogluconolactone. G6PD deficiency is the most common enzyme deficiency in humans affecting over 400 million people worldwide [13]. Recent evidences have revealed the role of G6PD and PPP in regulating endothelial cells [14, 15, 16, 17]. However, limited studies have revealed the correlation between G6PD deficiency and hypoxia‐induced endothelial dysfunction [18, 19, 20].

*N*‐Acetylcysteine (NAC) functions as a precursor of glutathione and a scavenger of free radicals [21]. Its anti‐inflammatory property in the context of noxious stimulus has been well demonstrated [22, 23, 24, 25]. Increasing evidences reported protective effects of NAC on endothelial cells. NAC treatment ameliorated diabetic retinopathy by reducing retinal pericyte loss, macrophage activation, lipid peroxidation, and scavenging ROS in high glucose‐treated bovine retinal vascular endothelial cells [26, 27, 28, 29] NAC can attenuate systemic platelet activation and cerebral vessel thrombosis in diabetes by increasing GSH level [30]. The capacity of NAC to accelerate the healing of amputation stump in diabetes and ischemia has also been reported, further implying the potential of NAC against hypoxia [31]. Furthermore, G6PD activity in gastric ulcers [25] and erythrocytes in lead‐exposed populations could be rescued by NAC treatment [32], indicating a correlation between G6PD and NAC. As a GSH precursor, NAC may replenish the lack of GSH caused by G6PD deficiency. Therefore, it is important to determine whether NAC treatment could compensate for any cell damage caused by G6PD deficiency as a therapeutic approach.

Therefore, in this study, we used the hypoxia‐mimetic agent cobalt chloride (CoCl_2_) to better elucidate mechanisms mediating endothelial dysfunction in the hypoxia‐related vascular diseases. Hypoxia‐inducible factor‐1 (HIF‐1) plays a crucial role in the adaptation of cells to hypoxia. Under normoxia, HIF‐1α modified by prolyl hydroxylases (PHD) can bind to the tumor suppressor von Hippel–Lindau (VHL) protein and be subsequently degraded by the proteasome. In the absence of oxygen, prolyl hydroxylation is blocked, so CoCl_2_ can stabilize HIF to mimic hypoxia by binding to the iron‐binding domain of HIF hydroxylase to prevent hydroxylation [33]. Thus, the involvement of G6PD and the protective effect of NAC on endothelial cells were further determined in the presence or absence of CoCl_2_ treatment. In summary, we identified G6PD as a novel molecular target in both arterial and venous endothelial cells treated with CoCl_2_. Moreover, the therapeutic potential of NAC to attenuate hypoxia‐induced vasculature lesions was indicated which could be mediated via an increase in G6PD activity.

## Results

### Treatment with CoCl_2_ caused endothelial dysfunction

**Fig. 1:**
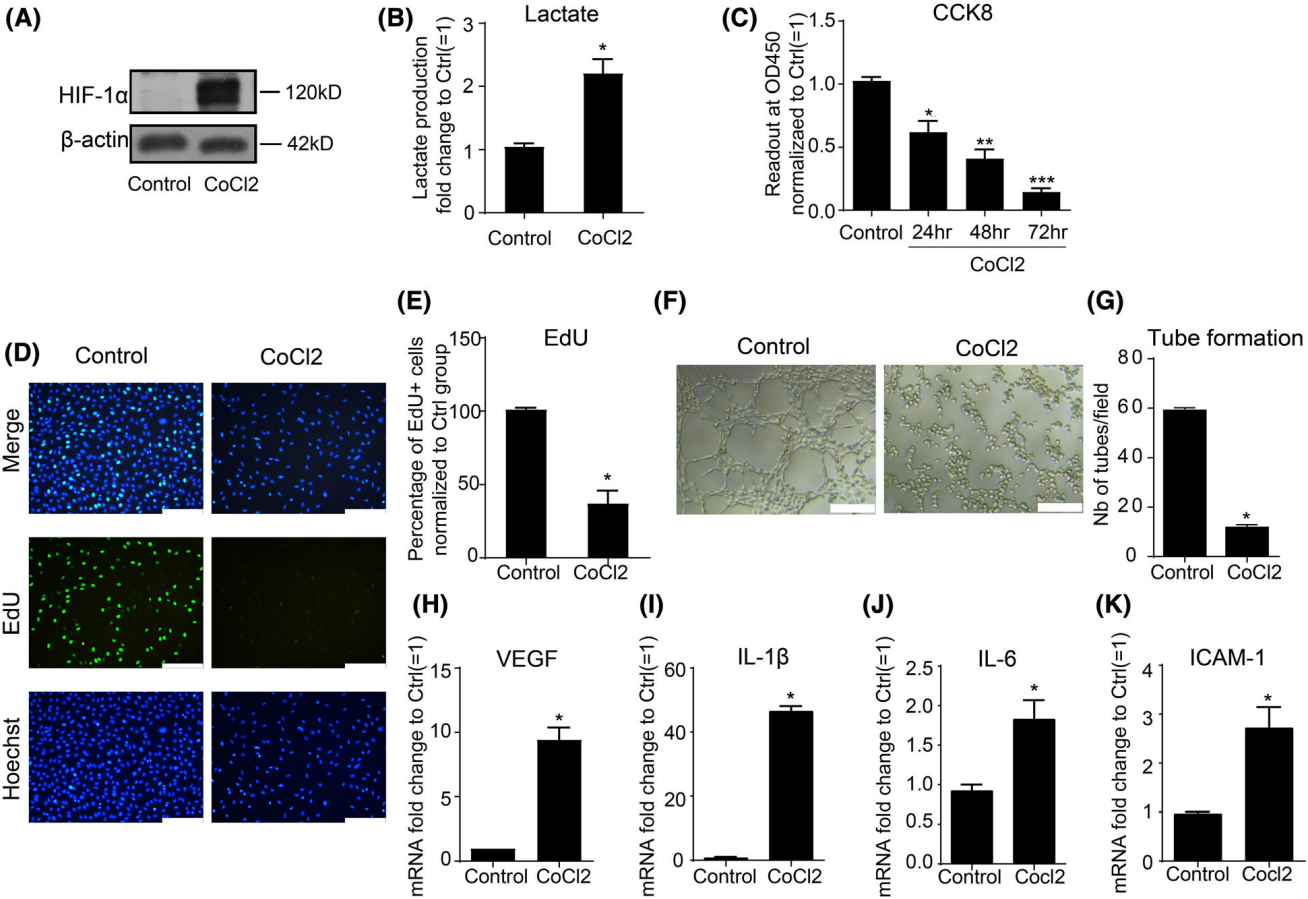
Treatment with CoCl_2_ caused endothelial dysfunction. (A,B) EA.hy926 endothelial cells treated with CoCl_2_ (400 μm) showed accumulation of HIF‐1α (120 kD) and lactate production; (C) cell viability was measured with CCK‐8 assay; (D,E) EdU assay was applied to evaluate the proliferation ability of endothelial cells upon CoCl_2_ treatment for 48 h at 400 μm. Scale bars = 200 μm; (F,G) Matrigel assay was applied to evaluate the tube formation capacity of endothelial cells upon 400 μm CoCl_2_ treatment for 48 h (right panel) compared to control group (left panel). Scale bars = 200 μm; (H–K) Real‐time PCR was applied to measure gene expression of VEGF, IL‐1β, IL‐6, ICAM‐1 in EA.hy926 cells stimulated with 400 μm CoCl_2_ for 48 h. Data are expressed as mean ± SEM of independent experiments (*n* ≥ 3). Student's *t*‐test or One‐way ANOVA test followed by Tukey's multiple comparison test was used. *P < 0.05, **P < 0.01, and ***P < 0.001 vs control group. [Colour figure can be viewed at wileyonlinelibrary.com]

A hybrid human vein endothelial cell line, EA.hy 926, was first exposed to CoCl_2_ treatment to establish an *in vitro* model that mimiced the hypoxic microenvironment. HIF‐1α accumulation was measured to confirm the hypoxic effect, which was further supported by lactate accumulation (Fig. 1A,B). CoCl_2_ treatment significantly reduced the cell viability in a time‐dependent manner (Fig. 1C). Simultaneously, reduced cell proliferation (Fig. 1D,E) and tube‐forming capacities (Fig. 1F,G) were observed after 48 h of CoCl_2_ treatment. Furthermore, the expression of classical inflammatory factors (IL‐1β, IL‐6, ICAM‐1) and the pro‐angiogenic factor VEGF were significantly increased in endothelial cells upon exposure to CoCl_2_ (Fig. 1H–K). Therefore, these results suggest that CoCl_2_‐induced hypoxic effect can cause endothelial cell dysfunction by reducing cell viability, proliferation, tube formation, and inducing cytokine secretion.

### Treatment with CoCl_2_ inhibited G6PD expression and activity

**Fig. 2:**
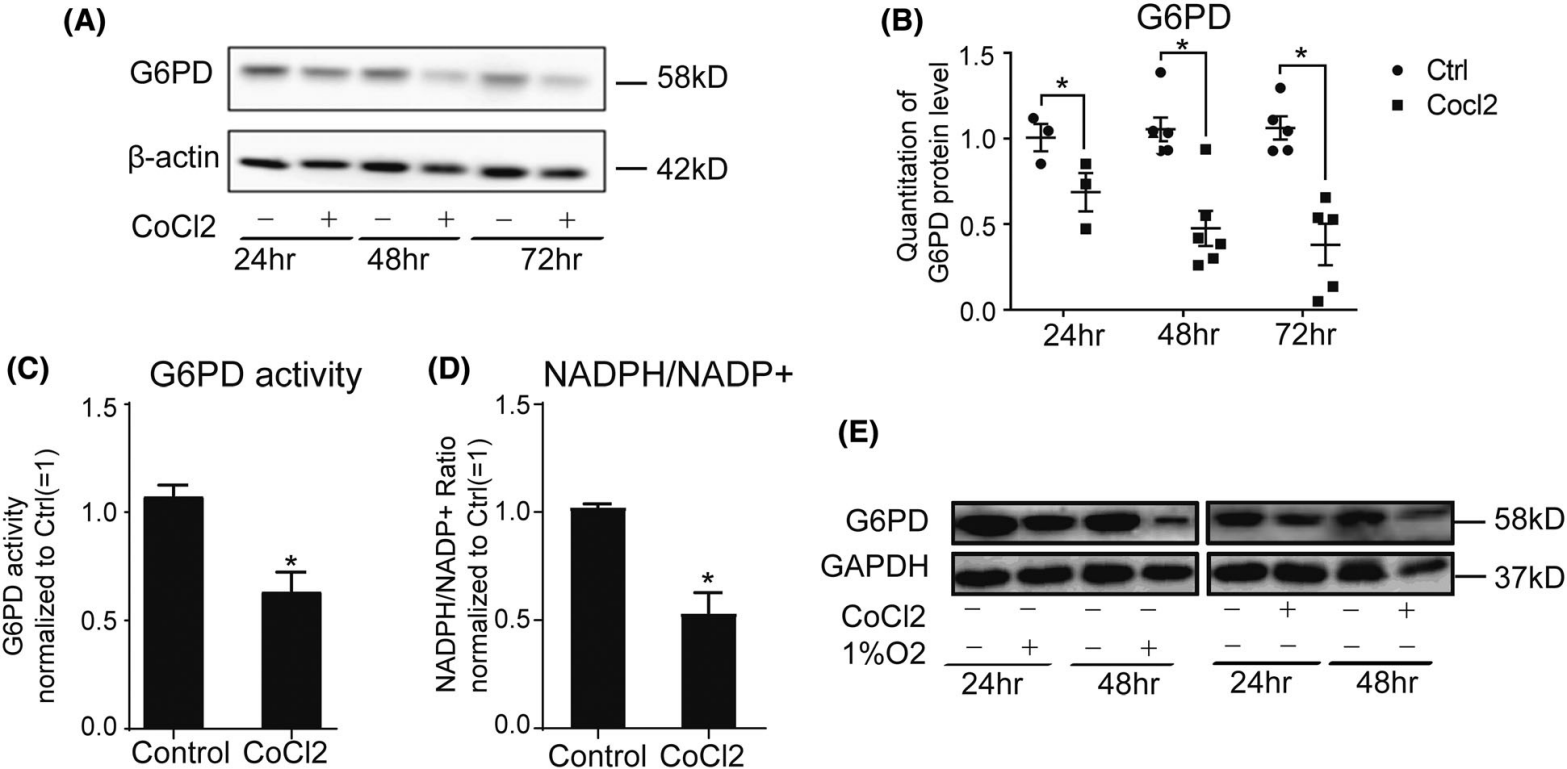
Treatment with CoCl_2_ inhibited G6PD expression and activity. (A,B) Treatment of EA.hy926 cells with CoCl_2_ at 400 μm time‐dependently inhibited G6PD (58 kD) expression; (C,D) G6PD activity, and products were inhibited by CoCl_2_ (400 μm) treatment for 48 h as determined by G6PD activity Assay kit and NADP/NADPH Assay kit; (E) Low oxygen (1%) stimulation for 24 and 48 h inhibited G6PD expression in EA.hy926 cell line, which was comparable to CoCl_2_ treatment at 400 μm. Data are expressed as mean ± SEM of independent experiments (*n* ≥ 3). Student's *t*‐test was used. *P < 0.05 vs control group.

To determine the involvement of G6PD, we measured G6PD expression and activity in endothelial cells exposed to hypoxia. It was noted that G6PD expression was time‐dependently inhibited upon CoCl_2_ treatment, as quantified (Fig. 2A,B). Subsequently, both G6PD enzyme activity and the ratio of NADPH/NADP^+^ significantly reduced in endothelial cells treated with CoCl_2_ for 48 h (Fig. 2C,D). The decreased G6PD expression in EA.hy926 cell upon exposure to CoCl_2_ was further supported by low oxygen (1%) stimulation for 24 and 48 h, comparable to CoCl_2_ treatment (Fig. 2E). In summary, these results suggest that G6PD expression and activity in EA.hy926 cells were attenuated by hypoxic stimulation induced by CoCl_2_.

### Pentose phosphate pathway is involved in endothelial dysfunction

**Fig. 3:**
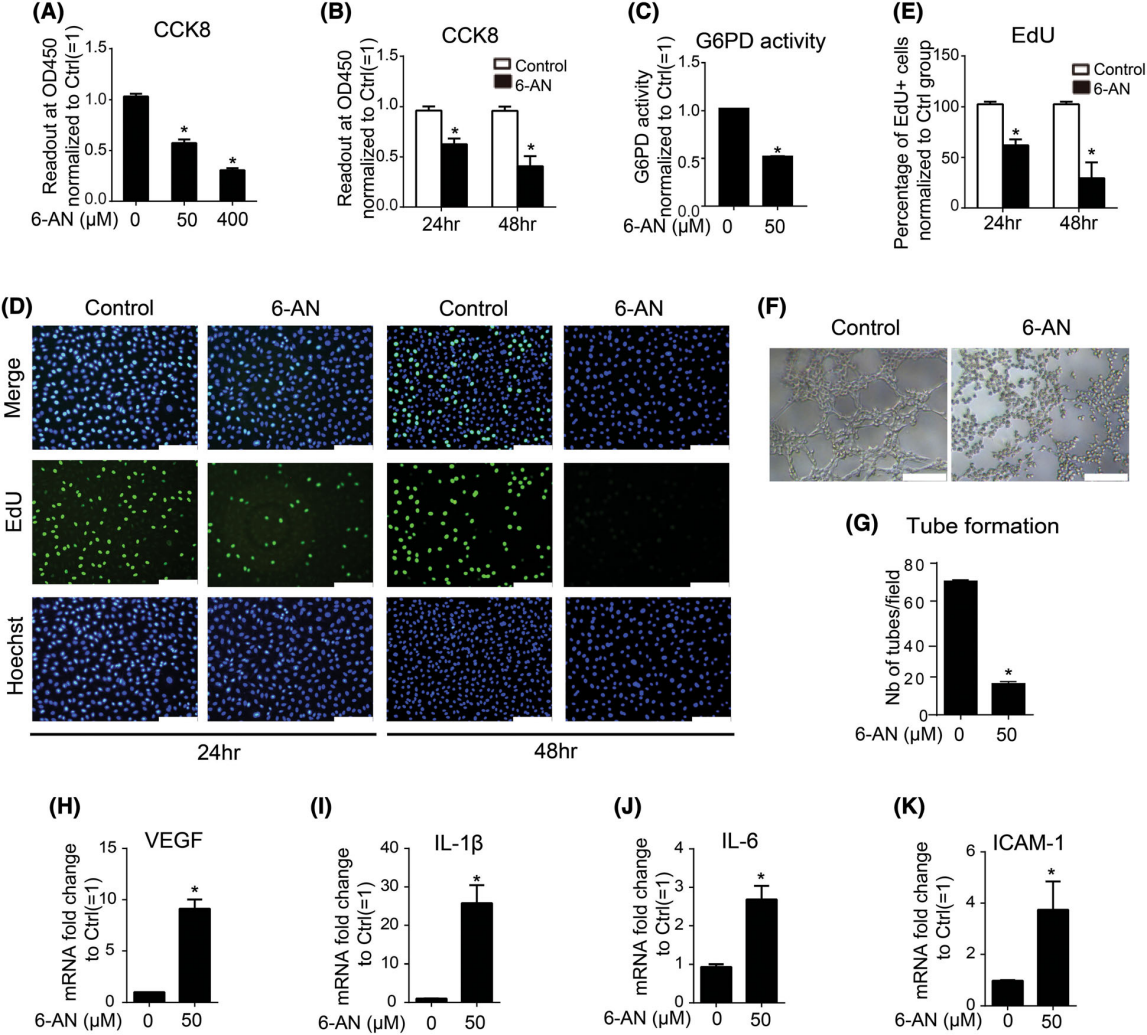
Pentose phosphate pathway is involved in endothelial dysfunction. (A,B) Addition of 6‐AN to EA.hy 926 cells time‐ and dose‐dependently decreased cells viability according to CCK‐8 assay; (C) Addition of 6‐AN at 50 μm for 48 h inhibited G6PD activity as measured by G6PD activity assay kit; (D,E) Addition of 6‐AN at 50 μm for 24 or 48 h inhibited cell proliferation according to EdU assay. Scale bars = 200 μm; (F and G) Addition of 6‐AN at 50 μm for 48 h inhibited tube formation. Scale bars = 200 μm; (H–K) Treatment with 6‐AN at 50 μm for 48 h induced activation of VEGF, IL‐1β, IL‐6, ICAM‐1, respectively. Data are expressed as mean ± SEM of independent experiments (*n* ≥ 3). Student's *t*‐test or One‐way ANOVA test followed by Tukey's multiple comparison test was used. *P < 0.05 vs control group. [Colour figure can be viewed at wileyonlinelibrary.com]

To determine whether G6PD‐mediated PPP is directly involved in causing endothelial damage, 6‐AN, a specific PPP inhibitor, was applied in this study to abrogate G6PD activity. 6‐AN was observed to inhibit endothelial cell viability in a dose‐ and time‐dependent manner (Fig. 3A,B). The ability of 6‐AN to inhibit G6PD activity was further confirmed at a dose of 50 μm (Fig. 3C). Consistently, cell proliferation was reduced upon 6‐AN treatment at the dose of 50 μm time‐dependently (Fig. 3D,E). Tube formation capacity determined by cell proliferation, survival, and migration abilities, was also abrogated by 6‐AN treatment for 48 h (Fig. 3F,G). Furthermore, the expression of angiogenic and inflammatory factors including VEGF, IL‐1β, IL‐6, and ICAM‐1, was directly activated by 6‐AN to inhibit G6PD activity and PPP (Fig. 3H–K). In summary, the results suggest that PPP inhibition by 6‐AN may cause endothelial dysfunction by hampering cell viability, proliferation, tube formation, and activation of inflammatory/angiogenic factors.

### NAC ameliorated CoCl_2_ ‐induced endothelial cell dysfunction and enhanced G6PD activity

**Fig. 4:**
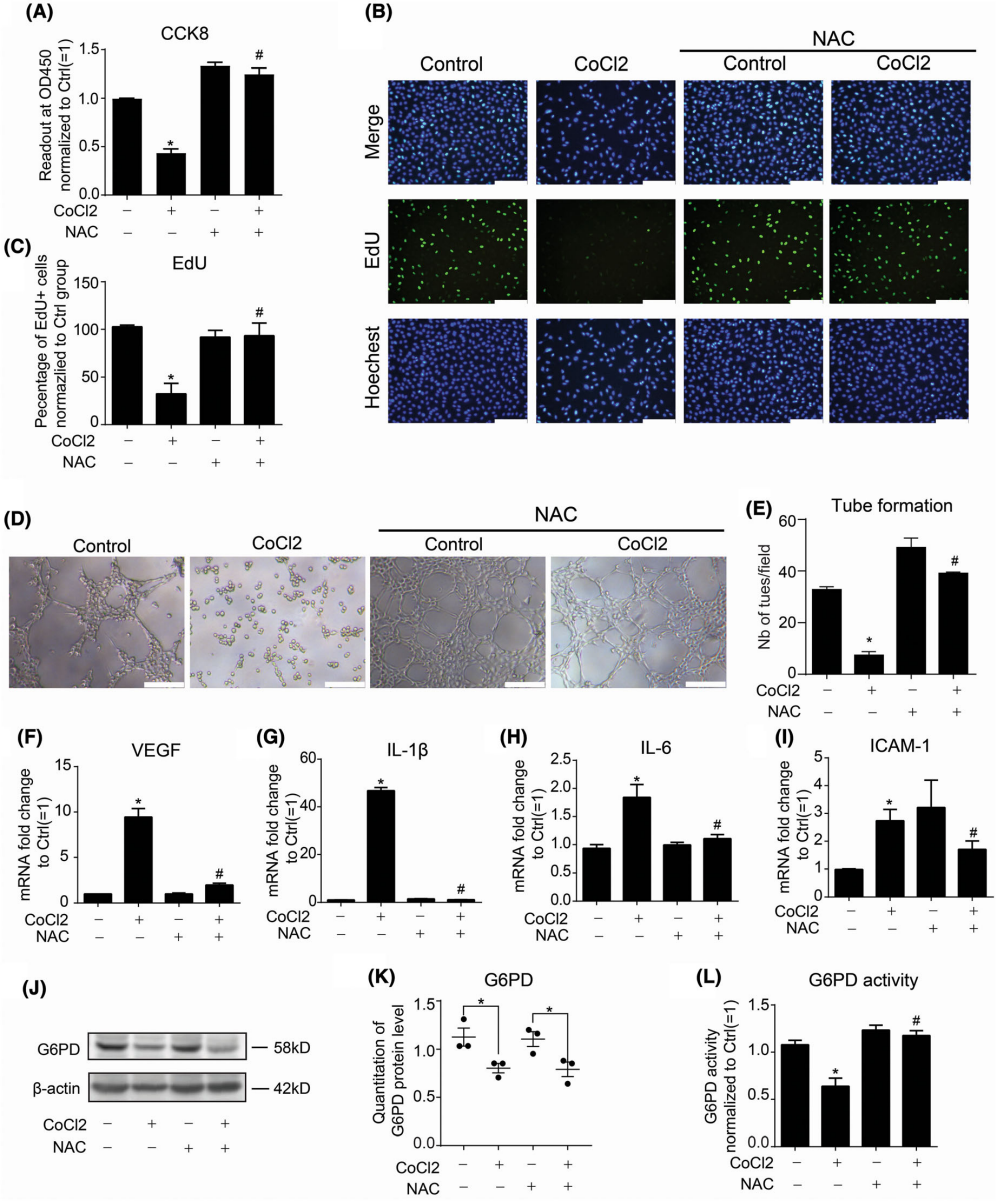
NAC ameliorated CoCl_2_ induced endothelial cell dysfunction. (A–E) Cell viability, proliferation, and tube formation capacity were measured by CCK‐8 assay, EdU assay, and matrigel tube formation in EA.hy926 cells stimulated with 400 μm CoCl_2_ for 48 h in the presence or absence of NAC (10 mm) pretreatment for 1 h, respectively. Scale bars = 200 μm; (F–I) Real‐time PCR was applied to measure gene expression of VEGF, IL‐1β, IL‐6, and ICAM‐1 in EA.hy926 cells stimulated with 400 μm CoCl_2_ for 48 h pretreated with or without NAC (10 mm) for 1 h; (J,K) Pretreatment with NAC at 10 mm for 1 h did not rescue decreased G6PD protein level caused by 400 μm CoCl_2_ for 48 h; (L) Reduced G6PD activity by treatment of 400 μm CoCl_2_ for 48 h was efficiently rescued by pretreatment with NAC at 10 mm for 1 h. Data are expressed as mean ± SEM of independent experiments (*n* ≥ 3). Student's *t*‐test or One‐way ANOVA test followed by Tukey's multiple comparison test was used. *P < 0.05 vs control group; #*P* < 0.05 vs CoCl_2_ group. [Colour figure can be viewed at wileyonlinelibrary.com]

Following the identification of G6PD as a candidate for CoCl_2_‐induced endothelial dysfunction, we identified known chemicals that may ameliorate G6PD deficiency for therapeutic purposes. Based on previous studies that revealed the positive effect of NAC on G6PD activity and its replenish effect on GSH, we performed experiments to determine whether NAC could affect G6PD expression or activity. NAC pretreatment was applied prior to CoCl_2_ stimulation, and the endothelial cell viability, proliferation, tube formation capacity, and expression of inflammatory/angiogenic factors with or without CoCl_2_ treatment were measured. Exposure to CoCl_2_ for 48 h significantly reduced cell proliferation, viability, and tube forming, which was efficiently rescued by NAC pretreatment (Fig. 4A–E). In addition, CoCl_2_‐mediated upregulation of VEGF, IL‐1β, IL‐6, and ICAM‐1 was also significantly abrogated by NAC (Fig. 4F–I). Notably, NAC had little effect on the expression of G6PD (Fig. 4J,K); however, it significantly rescued the G6PD activity after CoCl_2_‐incued reduction (Fig. 4L). In summary, these data suggest that NAC could ameliorate CoCl_2_‐induced cell damage, which may be dependent on enhanced G6PD activity.

### Protective effects of NAC against CoCl_2_ _−_induced cell damage is pentose phosphate pathway dependent

**Fig. 5:**
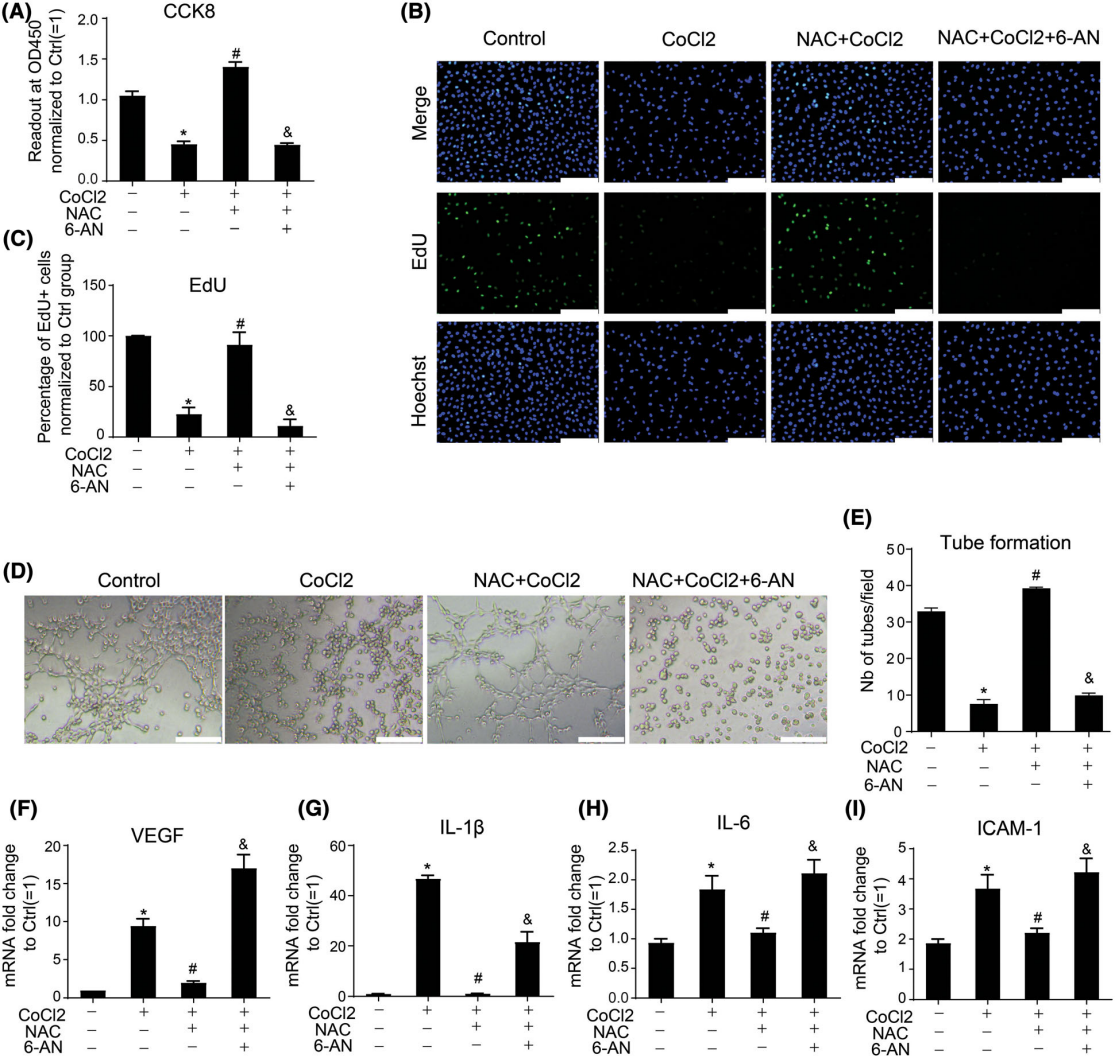
6‐AN abrogated the protective effect of NAC on endothelial cell damage induced by CoCl_2_. EA.hy926 endothelial cells were treated with CoCl_2_ in the presence or absence of NAC (10 mm) or 6‐AN (50 μm) pretreatment for 1 h. Cell viability, proliferation, tube formation capacity, and cytokine expression were measured by CCK‐8 assay(A), EdU assay (B,C), matrigel assay (D,E), and Real‐time PCR (F,I) in EA.hy926 cells stimulated with 400 μm CoCl_2_ for 48 h, respectively. Scale bars = 200 μm. Data are expressed as mean ± SEM of independent experiments (*n* ≥ 3). Student's *t*‐test or One‐way ANOVA test followed by Tukey's multiple comparison test was used. *P < 0.05 vs control group, #*P* < 0.05 vs CoCl_2_ group, &*P* < 0.05 vs NAC + CoCl_2_ group. [Colour figure can be viewed at wileyonlinelibrary.com]

Based on the rescue effect of NAC on G6PD activity, we further treated cells with NAC in the presence or absence of 6‐AN to confirm whether the protective effect of NAC was PPP dependent. As expected, 6‐AN significantly reduced the protective effect of NAC against CoCl_2_‐induced endothelial dysfunction, including cell viability, proliferation, and tube formation capacity (Fig. 5A–E). Furthermore, the inhibitory effect of NAC on CoCl_2_‐induced pro‐inflammatory/adhesion cytokine secretion was further attenuated by 6‐AN treatment (Fig. 5F–I). In addition to the EA.hy926 cell line, we also used primary human aortic endothelial cells (HAECs) to confirm the protective effects of NAC against CoCl_2_ treatment and involvement of PPP. Consistent with vein endothelial cells, CoCl_2_ treatment significantly reduced HAEC viability, proliferation, and tube formation capacities (Fig. S1A–E). Cytokines, including VEGF, IL‐1β, IL‐6, and ICAM‐1, were significantly upregulated (Fig. S1F–I). NAC ameliorated CoCl_2_‐induced the arterial endothelial dysfunction. Further addition of 6‐AN blunted the protective effects of NAC against CoCl_2_. In summary, these results suggest that G6PD activity and PPP mediates the protective effect of NAC in both venous and arterial endothelial cells against CoCl_2_ treatment.

### Phenotypic changes induced in vascular smooth muscle cells upon exposure to conditional medium from CoCl_2_ ‐treated endothelial cells is ameliorated by NAC and is pentose phosphate pathway dependent

Endothelial cells directly contact with VSMCs or pericytes to form barrier and regulate vessel functions. In response to external stimulus, VSMCs undergo a phenotypic change from a quiescent to a proliferative phenotye characterized by increased proliferation, migration, and secretion of pro‐inflammatory cytokines accompanied with increased expression of IL‐6, iNOS, and MMP9.

**Fig. 6:**
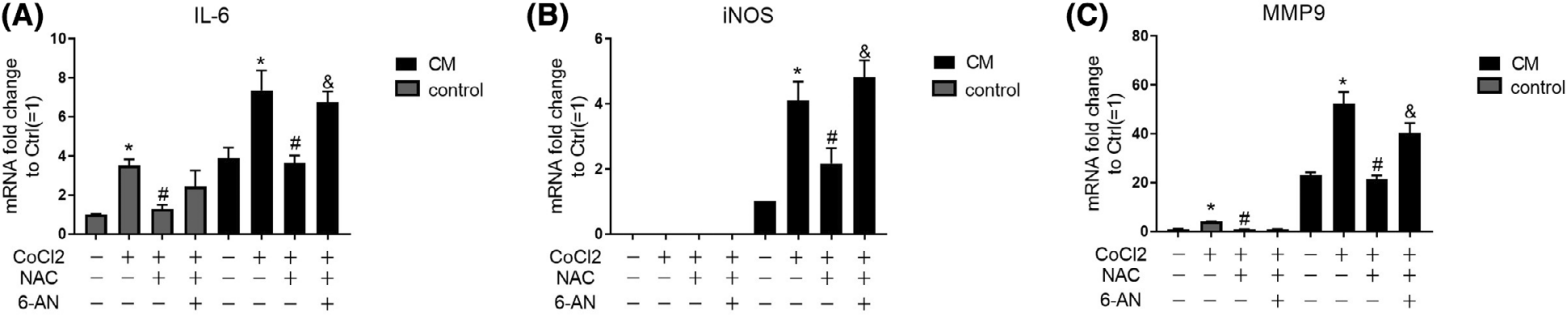
Synthetic phenotypic change of VSMCs induced by conditional medium from CoCl_2_‐treated endothelial cells is ameliorated by NAC which is G6PD dependent. mRNA level of IL‐6 (A), iNOS (B), and MMP9 (C) was evaluated in A7r5 rat aortic VSMC cell line treated with conditional medium (CM, black bars) from HAECs treated by CoCl_2_ in the presence or absence of NAC or 6‐AN for 48 h, or in fresh medium (control, gray bars) supplemented with CoCl_2_ (400 μm), NAC (10 mm), or 6‐AN (50 μm) for 24 h. Data are expressed as mean ± SEM of independent experiments (*n* = 3). One‐way ANOVA test followed by Tukey's multiple comparison test was used. *P < 0.05 vs control group, #*P* < 0.05 vs CoCl_2_ group, &*P* < 0.05 vs NAC + CoCl_2_ group. [Colour figure can be viewed at wileyonlinelibrary.com]

Furthermore, in this study, we evaluated how VSMCs respond to cytokines secreted by CoCl_2_‐treated endothelial cells. Compared to VSMCs cultured in fresh medium supplemented with CoCl_2_, NAC, or 6‐AN (control panel), VSMCs cultured in a conditioned medium from HAECs treated with CoCl_2_, NAC, or 6‐AN (CM panel) expressed higher levels of IL‐6, iNOS, and MMP9 (Fig. 6A–C). Moreover, compared to VSMCs cultured in a conditioned medium from HAECs with no further addition of CoCl_2_, NAC, or 6‐AN, VSMCs cultured in a conditioned medium from CoCl_2_‐treated HAECs exhibited significantly higher IL‐6, MMP9, and iNOS production, indicating the induction of pro‐inflammatory and migratory phenotypic changes in VSMCs. Notably, enhanced IL‐6, MMP9, and iNOS levels were blunted in VSMCs cultured in conditioned medium from CoCl_2_‐treated HAECs pretreated with NAC. Finally, the addition of 6‐AN to HAECs treated with CoCl_2_ and NAC resulted in recovered capacity of the HAECs conditioned medium to induce the expression of IL‐6, iNOS, and MMP9 in VSMCs (Fig. 6A–C). In summary, these results indicate that synthetic phenotypic changes in VSMCs could be induced by secreted cytokines from CoCl_2_‐treated endothelial cells, which can be ameliorated by NAC and is G6PD activity/PPP dependent.

## Discussion

Acute or chronic hypoxia and ischemia have been widely observed in many vascular‐related disorders, including diabetic vascular complications like diabetic retinopathy or kidney disease, chronic obstructive pulmonary disease, obstructive sleep apnea, atherosclerosis, and myocardial ischemia reperfusion injury (I/RI). As the first layer in the lumen of vessels, endothelial cells are the first cell population to sense oxygen changes in the vasculature, indicating that endothelial cells may serve as a potential target for the treatment of hypoxia‐related vascular diseases. Our data identified G6PD as a novel target for CoCl_2_‐induced endothelial dysfunction. Furthermore, this study demonstrated the protective effect of NAC against CoCl_2_‐induced endothelial dysfunction by enhancing G6PD activity, supporting its therapeutic potential.

To date, studies on the role of G6PD in vascular diseases, particularly those related to hypoxia, remain limited. Diabetic retinopathy (DR), including nonproliferative DR, proliferative DR, and macular edema, is closely related to hypoxia. It was found that oxygen therapy in patients with chronic diabetic macular edema could significantly reduce retinal thickness, indicating the involvement of hypoxia [34]. Recent clinical evidence has demonstrated the incidence of G6PD deficiency in concert with proliferative diabetic retinopathy (PDR) which is more likely to occur in patients with type I diabetes mellitus over 15 years with G6PD deficiency [18]. In addition, decreased erythrocyte G6PD activity and low NADPH levels have been detected during the early phase of nonproliferative diabetic retinopathy (NPDR) with increased advanced lipoxidation end product (ALE) generation [35]. Consistently, decreased G6PD expression and activity upon CoCl_2_ treatment in endothelial cells was observed in this study.

The effects of hypoxia on G6PD expression and activity remain controversial and largely unknown. G6PD expression is enhanced in response to hypoxia (3% O_2_) and this results in a switch in pulmonary artery smooth muscle cell phenotype, that is, from the contractile to synthetic phenotype, thereby contributing to pulmonary arterial remodeling and pathogenesis of pulmonary hypertension [36]. Hypoxia (3% O_2_) or CoCl_2_ (100 μm) also activated G6PD expression and activity in PC12 cells in a ROS‐dependent manner [37]. In contrast, in vascular smooth muscle cells, hypoxia reduced the PPP flux, leading to decreased NADPH levels and, therefore, increased vasorelaxation [38], which is consistent with the findings of our study in endothelial cells. Considering the discrepancies in the effects of hypoxia on G6PD, we speculated that the regulation of G6PD is dependent on the metabolic characteristics of the type of cell, degree of hypoxia, and extent of ROS accumulation. In this study, no accumulation of ROS was detected in endothelial cells upon CoCl_2_ stimulation for either 24 or 48 h (Fig. S2A). To better elucidate this, we measured the expression of key Nrf2/ARE genes that regulate the antioxidative defense system, including *CAT* (Catalase), *HO‐1* (heme oxygenase 1), *GPX1* (glutathione peroxidase 1), *GSS* (glutathione synthetase), *GSR* (glutathione‐disulfide reductase), *NQO1* (NAD(P)H quinone dehydrogenase 1), *SOD1* (superoxide dismutase 1), and *SOD2* (superoxide dismutase 2; Fig. S2B). Interestingly, all the above genes were activated upon CoCl_2_ treatment indicating an activation of the antioxidative defense system, which may explain why no ROS accumulation was observed in EA.hy 926 in response to CoCl_2_. Moreover, supplementation with NAC, a well‐known ROS scavenger, did not rescue G6PD expression upon CoCl_2_ stimulation, as shown in Fig. 4, which strongly suggested that the decreased G6PD expression upon CoCl_2_ stimulation was not caused by ROS accumulation. Of interest, in contrast to protein level, G6PD mRNA was moderately increased upon CoCl_2_ (Fig. S3A). Therefore, it is indicated that G6PD protein reduction upon CoCl_2_ in this study was due to increased protein degradation. It is known that G6PD was also a key antioxidant gene responsible for NADPH synthesis and activated by Keap‐Nrf2 system. Thus, we speculated that the upregulation of G6PD mRNA may be mediated by Keap‐Nrf2 system.

In addition, the preference of endothelial cells for glycolysis, especially during oxygen shortage has been well presented by Carmeliet's group about the adaption capacity of endothelial cells oxygen shortage [39, 40], which was further supported by Jarmuszkiewicza's group that reported a shift from aerobic to anaerobic catabolism in response to hypoxia stimulation [41]. Furthermore, a metabolic switch from PPP to glycolysis was suggested in Kathagen–Buhmann's research demonstrating hypoxia‐induced downregulation of PPP enzymes concomitant with the upregulation of glycolysis enzymes [42]. In line with the findings, a metabolic shift toward glycolysis was observed in this study, as indicated by the increased levels of glycolysis product lactate in endothelial cells upon treatment with 400 μm CoCl_2_. Consistently, the apoptosis assay using Annexin/PI double staining showed no increase in early or late apoptosis of cells treated with CoCl_2_ (Fig. S4A,B). Thus, it may be postulated that endothelial cells adapt to the hypoxic microenvironment by diverting carbon away from mitochondrial respiration to glycolysis to avoid ROS formation. In consistent with this, we have done the dose test of CoCl_2_ using CCK8 assay and noticed that in compared to most other studies, higher dose of CoCl_2_ (400 μm) was required to induce significant endothelial cell damage. This phenomenon may further support the adaption capacity of endothelial cells to hypoxia (Fig. S3B). It is important to identify the mechanism mediating G6PD inhibition upon hypoxia or CoCl_2_ treatment, independent of ROS, in future studies.

Our data showed that the protective effect of NAC against CoCl_2_ mimicked the hypoxic effect by enhancing G6PD activity, but not expression. As a thiol‐containing compound, NAC has been widely applied clinically for decades to treat acetaminophen toxicity [43], contrast‐induced nephropathy, chronic obstructive pulmonary disorder, and pulmonary fibrosis [44, 45]. In addition, clinical application of NAC in the treatment of other chronic diseases, such as inflammatory bowel disease, has also been reported [46]. Thus, NAC may serve as a potential therapy for treating hypoxia‐related endothelial dysfunction in the future. NAC supplementation to compensate G6PD deficiency have been reported in several diseases [25, 32, 47]. As a GSH precursor, NAC mostly functions to combat oxidative stress by increasing the levels of GSH, which could also be catalyzed by G6PD from GSSG. Therefore, treatment with NAC could replenish the lack of GSH caused by G6PD deficiency as reported in several studies. However, it is worth noting that the addition of 6‐AN abrogated NAC treatment in this study, suggesting that PPP is indispensable for the protective effect of NAC against hypoxia. Thus, instead of replenishing GSH, it is rational to assume that NAC protects endothelial cells against CoCl_2_ treatment by activating G6PD activity to produce ribose‐5‐phosphate and NADPH for biosynthetic processes. To date, several mechanisms involved in modulating G6PD activity post‐translation have been revealed, including O‐GlcNAcylation and phosphorylation [9, 10, 48, 49, 50]. Therefore, it is necessary to investigate how NAC regulates G6PD activity in future study.

In this study, CoCl_2_ was applied to prevent the HIF protein degradation and induce hypoxia effect. However, HIF‐1‐independent effects has also been reported. It was found that upon acute hypoxia stimulation by oxygen shortage, monocyte adhesion to endothelial cells was induced by increased ICAM‐1 expression. Moreover, ICAM‐1 upregulation was mediated by prolyl hydroxylase (PHD)‐dependent NF‐κB activation, not HIF‐1 or HIF‐2. In consistent with this, cross‐talk between hypoxia and inflammation has been well revealed. In consideration of activation of inflammatory cytokines upon CoCl_2_ in this study, it would be interesting to knock down HIF and PHD separately to elucidate the underlying mechanisms mediating CoCl_2_ treatment and endothelial dysfunction, which can be either HIF‐dependent or ‐independent [8, 51].

Finally, it is necessary to emphasize that this study was based on the *in vitro* model, which cannot exactly reflect *in vivo* situation. For example, endothelial cell proliferation capacity was limited *in vivo*. Although cell viability, proliferation, tube formation, and activation of inflammatory/angiogenic cytokine were analyzed in this study, we speculated that compared to proliferation and tube‐forming capacities, cell viability and inflammatory cytokine secretion would be more relevant to the *in vivo* situation and worthy for further investigation in the future.

## Conclusions

In conclusion, our study identified G6PD as a novel candidate in CoCl_2_‐induced endothelial cell damage, including abrogated cell viability, proliferation, tube formation, and activation of pro‐inflammatory/angiogenetic cytokines. Furthermore, pretreatment with NAC could ameliorate CoCl_2_‐induced endothelial cell damage by activating G6PD activity, indicating its therapeutic potential in treating hypoxia‐related endothelial dysfunction.

## Materials and methods

### Cell culture

EA.hy926 (ATCC® CRL‐2922™, Gaithersburg, MD, USA) cell line was a immobilized human umbilical vein cell line established by fusing primary human umbilical vein cells with a thioguanine‐resistant clone of A549 and cultured in DMEM (5.5 mm d‐glucose) supplemented with 10% FBS and 1% penicillin/streptomycin in a humidified atmosphere of 5% CO_2_ at 37 °C. HAECs(A kind gift from Dr Wei Zhang, Procell, China) and A7r5 (Procell, Wuhan, China) were cultured in DMEM (25 mm d‐glucose) supplemented with 10% FBS and 1% penicillin/streptomycin. HAECs were primary human aortic endothelial cells isolated from the human ascending and descending aorta. Passages at 4–9 were applied *in vitro* culture for HAECs and A7r5. Endothelial cells were passaged at ∼ 90% confluence and seeded at 2 × 10^4^ cells·cm^−2^, which normally become 100% confluent without further treatment after 48 h. NAC (10 mm) and 6‐AN (50 μm) were added 1 h prior to CoCl_2_ (400 μm) treatment.

For conditional medium experiment, HAECs were treated with CoCl_2_ at 400 μm in the presence or absence of NAC (10 mm) or 6‐AN (50 μm) for 48 h. Afterward, HAEC culture media from four groups (control, CoCl_2_, CoCl_2_ + NAC, and CoCl_2_ + NAC + 6‐AN) was harvested and quickly spun to remove cell debris. Afterward, these conditional media from four groups were added directly to confluent A7r5 rat aortic VSMCs for another 24 h. A7r5 cells were then harvested for qPCR analysis for IL‐6, iNOS, and MMP9 mRNA level. A7r5 cells were also treated directly with CoCl_2_, NAC, or 6‐AN and compared to those treated with conditional medium from HAECs.

### Real‐time polymerase chain reaction (PCR)

RNA was extracted with RNA simple Total RNA Kit (Tiangen Biotech, Beijing, China). cDNA synthesis was performed with the FastQuant RT Kit (Tiangen Biotech) following the manufacturer's instruction. Real‐time quantitative PCR was carried out with the SuperReal PreMix Plus (Tiangen Biotech) in a Light Cycler 480 System (Roche, Shanghai, China) or QuantStudio™ 7 Flex Real‐Time PCR System. Sequence of primers were listed in Table 1. The ΔΔ*C*_t_ method was applied to calculate relative expression level of target genes to endogenous control‐β‐actin.

**Table 1:** Sequences of primers for real‐time PCR.

Species	Gene symbol	Accession number	Forward primer (5′–3′)	Reverse primer(5′–3′)
Rat	*β‐ACTIN*	NM_031144	CGGTCAGGTCATCACTATCG	TTCCATACCCAGGAAGGAAG
Rat	*iNOS*	NM_012611.3	GGGGACTGGACTTTTAGAGACG	TGCACCAACTCTGCTGTTCTC
Rat	*MMP9*	NM_031055.2	TTCAAGGACGGTCGGTATT	CTCTGAGCCTAGACCCAACTTA
Rat	*IL‐6*	NM_012589.2	CATTCTGTCTCGAGCCCACC	GCTGGAAGTCTCTTGCGGAG
Hu	*VEGF*	NM_001287044.1	TCAAGCCATCCTGTGTGCC	GGCCTTGGTGAGGTTTGATCC
Hu	*IL‐1β*	NM_000576	AGCTACGAATCTCCGACCAC	CGTTATCCCATGTGTCGAAGAA
Hu	*β‐ACTIN*	NM_001101	ATTGGCAATGAGCGGTTCC	GGTAGTTTCGTGGATGCCACA
Hu	*IL‐6*	NM_000600	AGTGAGGAACAAGCCAGAGC	AGCTGCGCAGAATGAGATGA
Hu	*ICAM‐1*	NM_000201.2	GTATGAACTGAGCAATGTGCAAG	GTTCCACCCGTTCTGGAGTC

### Western blot

Protein samples were loaded at 10 μg per lane, separated by 10% SDS‐polyacrylamide gel electrophoresis, and then transferred to poly vinylidene fluoride membrane. Membranes were probed with anti‐G6PD antibody(1 : 1000, Abcam, Shanghai, China), anti‐β‐actin, or GAPDH antibody (1 : 1000, Sigma‐Aldrich, Shanghai, China) overnight at 4 °C then incubated with Horseradish‐conjugated anti‐rabbit or mouse antibody (1 : 5000, Pierce, Thermofisher, Waltham, MA, USA) before development in ECL substrate and auto‐radiographed using imagequant las 4000 mini (Cytiva, Shanghai, China).

### NADP/NADPH assay

The NADP/NADPH ratio was measured using a NADP/NADPH assay kit (Abcam, ab65349, Cambridge, UK) according to the manufacturer's instructions. Briefly, cells harvested from a 10 cm dish were immediately lysed in 400 μL of extraction buffer, followed by two freeze/thaw cycles (20 min on dry ice followed by 10 min at RT). The lysate was centrifuged at 13 000 × ***g*** for 5 min. Two‐hundred mictroliters of supernatant from each sample were heated at 60 °C for 30 min in a water bath and then measured by reaction with the reaction mixure. The optimal readouts were collected after 1 h at room temperature.

### G6PD activity

G6PD activity was measured utilizing a Glucose‐6‐Phosphate Dehydrogenase Activity Colorimetric Assay kit (Biovision, K757 or Abcam, ab102529) according to the manufacturer's instructions. Briefly, 1 × 10^6^ cells were rapidly homogenized in an equivalent volume of G6PDH Assay Buffer. Fifty microliters of lysis were added into a 96‐well plate with the addition of another 50 μL reaction mix. Absorbance at OD 450 nm before and after incubation at 37 °C for 30 min was recorded. G6PD activity was calculated using the NADH standard curve.

### Measurement of cell proliferation

Cell proliferation was measured using the Cell‐Light™ EdU Apollo®488 *In Vitro* Imaging Kit (Ribobio, C10310‐3, Guangzhou, China) according to the manufacturer's instructions. Briefly, cells were cultured for 2 h in a medium supplemented with 50 μm EdU before fixation with 4% paraformaldehyde for 30 min at RT. Cells were permeabilized with 0.5% Triton‐100 in PBS, stained with EdU staining buffer as instructed by the manufacturer, and counterstained with Hoechst33342 for nucleus. The cell proliferation rate was represented by the percentage of positively labeled cells among total cells and normalized to the control group (=100%).

### Measurement of cell viability

Cell viability was measured using a Cell Counting Kit‐8 (Dojindo, Kumamoto, Janpan) according to the manufacturer's instructions in a 96‐well plate. The culture medium was replaced to normal culture medium before adding the CCK8 solution. The absorbance of each well was determined at 450 nm and normalized to that of the control group (=1).

### Measurement of cell apoptosis and ROS accumulation

Cell apoptosis was measured using Annexin V, FITC Apoptosis Detection Kit (Dojindo) according to the manufacturer's instructions and analyzed using a flow cytometer (Annexin V, FITC: 494 nm/518 nm; PI: 535 nm/617 nm). ROS detection was measured using the CellROX™ Green kit (ThermoFisher, Shanghai, China) and then analyzed using a flow cytometer in the green channel (FITC channel).

### Measurement of tube formation capacity

Tube formation capacity was measured using Growth Factor Reduced Matrigel™ Matrix (354230; Corning, NY, USA) according to the manufacturer's instructions. Matrigel was thawed overnight on ice at 4 °C. EA.hy926 cells and HAECs were seeded in 6‐well plates and treated as indicated for 48 h. Fifty microliters of matrigel were seeded in a 96‐well plate per well and equilibrated to 37 °C for 30 min. Cells were harvested and seeded on matrigel at a density of 5 × 10^4^ cells. Tube formation was observed after 5 h under a microscope (TCS SP5, Leica, Wetzlar, Germany). Number of tubes was counted directly in 4–6 random fields.

### Statistical analysis

All data are presented as the mean ± SEM. Student's *t*‐test was used to compare the variation between two groups. Statistical analysis between more than two groups was performed using one‐way ANOVA followed by Tukey's multiple comparison test using the graphpad prism 7.0 software (GraphPad, San Diego, CA, USA). *P* < 0.05 was considered statistically different.

## Conflict of interest

The authors declare no conflict of interest.

## Author contributions

All authors participated in the design, interpretation of the studies, and analysis of the data and review of the manuscript. SY, SL, and CH conceived the idea, designed experiments, and reviewed the manuscript. CY and CH designed experiments and performed data analysis. CY, CH, XZ, and XG performed the experiments. CH and SL participated in data analysis. CY and CH drafted the manuscript. All authors have approved the submitted version.

## Supporting information


**Fig. S1.** 6‐AN abrogated the protective effect of NAC on arterial endothelial cell damage induced by CoCl_2_.
**Fig. S2.** CoCl_2_ treatment to mimic hypoxia effect did not induce ROS accumulation in EA.hy926 cells due to enhanced antioxidative defense system.
**Fig. S3.** G6PD mRNA change upon CoCl_2_ treatment and CoCl_2_ dose test.
**Fig. S4.** CoCl_2_ treatment to mimic hypoxia effect did not induce HAEC apoptosis.

## Data Availability

The datasets used and/or analyzed during this study are available from the corresponding author on reasonable request.

## References

[1] Li J , Wang JJ , Yu Q , Wang M , Zhang SX . Endoplasmic reticulum stress is implicated in retinal inflammation and diabetic retinopathy. FEBS Lett. 2009;583:1521–7. 19364508 10.1016/j.febslet.2009.04.007 PMC2691649

[2] Wang S , Wang C , Yan F , Wang T , He Y , Li H , et al. N‐Acetylcysteine attenuates diabetic myocardial ischemia reperfusion injury through inhibiting excessive autophagy. Mediators Inflamm. 2017;2017:9257291. 28265179 10.1155/2017/9257291 PMC5317145

[3] Costa PZ , Soares R . Neovascularization in diabetes and its complications. Unraveling the angiogenic paradox. Life Sci. 2013;92:1037–45. 23603139 10.1016/j.lfs.2013.04.001

[4] Braun L , Kardon T , Reisz‐Porszasz ZS , Banhegyi G , Mandl J . The regulation of the induction of vascular endothelial growth factor at the onset of diabetes in spontaneously diabetic rats. Life Sci. 2001;69:2533–42. 11693260 10.1016/s0024-3205(01)01327-3

[5] Ramakrishnan S , Anand V , Roy S . Vascular endothelial growth factor signaling in hypoxia and inflammation. J Neuroimmune Pharmacol. 2014;9:142–60. 24610033 10.1007/s11481-014-9531-7 PMC4048289

[6] Michiels C , Arnould T , Remacle J . Endothelial cell responses to hypoxia: initiation of a cascade of cellular interactions. Biochim Biophys Acta. 2000;1497:1–10. 10838154 10.1016/s0167-4889(00)00041-0

[7] Liu H , Wang Z , Yu S , Xu J . Proteasomal degradation of O‐GlcNAc transferase elevates hypoxia‐induced vascular endothelial inflammatory responsedagger. Cardiovasc Res. 2014;103:131–9. 24788415 10.1093/cvr/cvu116 PMC4133591

[8] Eltzschig HK , Carmeliet P . Hypoxia and inflammation. N Engl J Med. 2011;364:656–65. 21323543 10.1056/NEJMra0910283 PMC3930928

[9] Zhou L , Wang F , Sun R , Chen X , Zhang M , Xu Q , et al. SIRT5 promotes IDH2 desuccinylation and G6PD deglutarylation to enhance cellular antioxidant defense. EMBO Rep. 2016;17:811–22. 27113762 10.15252/embr.201541643 PMC5278614

[10] Tang HY , Ho HY , Wu PR , Chen SH , Kuypers FA , Cheng ML , et al. Inability to maintain GSH pool in G6PD‐deficient red cells causes futile AMPK activation and irreversible metabolic disturbance. Antioxid Redox Signal. 2015;22:744–59. 25556665 10.1089/ars.2014.6142 PMC4361223

[11] Zhang W , Ni C , Sheng J , Hua Y , Ma J , Wang L , et al. TLQP‐21 protects human umbilical vein endothelial cells against high‐glucose‐induced apoptosis by increasing G6PD expression. PloS ONE. 2013;8:e79760. 24278172 10.1371/journal.pone.0079760 PMC3836798

[12] Coffe V , Carbajal RC , Salceda R . Glucose metabolism in rat retinal pigment epithelium. Neurochem Res. 2006;31:103–8. 16475003 10.1007/s11064-005-9236-7

[13] Cappellini MD , Fiorelli G . Glucose‐6‐phosphate dehydrogenase deficiency. Lancet. 2008;371:64–74. 18177777 10.1016/S0140-6736(08)60073-2

[14] Afzal‐Ahmed I , Mann GE , Shennan AH , Poston L , Naftalin RJ . Preeclampsia inactivates glucose‐6‐phosphate dehydrogenase and impairs the redox status of erythrocytes and fetal endothelial cells. Free Radic Biol Med. 2007;42:1781–90. 17512457 10.1016/j.freeradbiomed.2007.02.032

[15] Leopold JA , Walker J , Scribner AW , Voetsch B , Zhang YY , Loscalzo AJ , et al. Glucose‐6‐phosphate dehydrogenase modulates vascular endothelial growth factor‐mediated angiogenesis. J Biol Chem. 2003;278:32100–6. 12777375 10.1074/jbc.M301293200

[16] Leopold JA , Zhang YY , Scribner AW , Stanton RC , Loscalzo J . Glucose‐6‐phosphate dehydrogenase overexpression decreases endothelial cell oxidant stress and increases bioavailable nitric oxide. Arterioscler Thromb Vasc Biol. 2003;23:411–7. 12615686 10.1161/01.ATV.0000056744.26901.BA

[17] Zhang Z , Apse K , Pang J , Stanton RC . High glucose inhibits glucose‐6‐phosphate dehydrogenase via cAMP in aortic endothelial cells. J Biol Chem. 2000;275:40042–7. 11007790 10.1074/jbc.M007505200

[18] Cappai G , Songini M , Doria A , Cavallerano JD , Lorenzi M . Increased prevalence of proliferative retinopathy in patients with type 1 diabetes who are deficient in glucose‐6‐phosphate dehydrogenase. Diabetologia. 2011;54:1539–42. 21380594 10.1007/s00125-011-2099-3

[19] Wilkinson‐Berka JL , Tan G , Jaworski K , Harbig J , Miller AG . Identification of a retinal aldosterone system and the protective effects of mineralocorticoid receptor antagonism on retinal vascular pathology. Circ Res. 2009;104:124–33. 19038868 10.1161/CIRCRESAHA.108.176008

[20] Pinna A , Contini EL , Carru C , Solinas G . Glucose‐6‐phosphate dehydrogenase deficiency and diabetes mellitus with severe retinal complications in a Sardinian population, Italy. Int J Med Sci. 2013;10:1907–13. 24324368 10.7150/ijms.6776 PMC3856382

[21] Mokhtari V , Afsharian P , Shahhoseini M , Kalantar SM , Moini A . A review on various uses of N‐Acetyl Cysteine. Cell J. 2017;19:11–7. 28367412 10.22074/cellj.2016.4872 PMC5241507

[22] Liu SC , Lee HP , Hung CY , Tsai CH , Li TM , Tang CH . Berberine attenuates CCN2‐induced IL‐1beta expression and prevents cartilage degradation in a rat model of osteoarthritis. Toxicol Appl Pharmacol. 2015;289:20–9. 26344001 10.1016/j.taap.2015.08.020

[23] Liu Y , Yao W , Xu J , Qiu Y , Cao F , Li S , et al. The anti‐inflammatory effects of acetaminophen and N‐acetylcysteine through suppression of the NLRP3 inflammasome pathway in LPS‐challenged piglet mononuclear phagocytes. Innate Immun. 2015;21:587–97. 25575547 10.1177/1753425914566205

[24] Park JH , Kang SS , Kim JY , Tchah H . The antioxidant N‐Acetylcysteine inhibits inflammatory and apoptotic processes in human conjunctival epithelial cells in a high‐glucose environment. Invest Ophthalmol Vis Sci. 2015;56:5614–21. 26305534 10.1167/iovs.15-16909

[25] Soliman NA , Zineldeen DH , Katary MA , Ali DA . N‐acetylcysteine a possible protector against indomethacin‐induced peptic ulcer: crosstalk between antioxidant, anti‐inflammatory, and antiapoptotic mechanisms. Can J Physiol Pharmacol. 2017;95:396–403. 28092180 10.1139/cjpp-2016-0442

[26] Tsai GY , Cui JZ , Syed H , Xia Z , Ozerdem U , McNeill JH , et al. Effect of N‐acetylcysteine on the early expression of inflammatory markers in the retina and plasma of diabetic rats. Clin Experiment Ophthalmol. 2009;37:223–31. 19723131 10.1111/j.1442-9071.2009.02000.x PMC3947378

[27] Zhou T , Zhou KK , Lee K , Gao G , Lyons TJ , Kowluru R , et al. The role of lipid peroxidation products and oxidative stress in activation of the canonical wingless‐type MMTV integration site (WNT) pathway in a rat model of diabetic retinopathy. Diabetologia. 2011;54:459–68. 20978740 10.1007/s00125-010-1943-1 PMC3017315

[28] Adachi T , Aida K , Nishihara H , Kamiya T , Hara H . Effect of hypoxia mimetic cobalt chloride on the expression of extracellular‐superoxide dismutase in retinal pericytes. Biol Pharm Bull. 2011;34:1297–300. 21804221 10.1248/bpb.34.1297

[29] Chen Y , Xu X , Sheng M , Zhang X , Gu Q , Zheng Z . PRMT‐1 and DDAHs‐induced ADMA upregulation is involved in ROS‐ and RAS‐mediated diabetic retinopathy. Exp Eye Res. 2009;89:1028–34. 19748504 10.1016/j.exer.2009.09.004

[30] Wang B , Yee Aw T , Stokes KY . N‐acetylcysteine attenuates systemic platelet activation and cerebral vessel thrombosis in diabetes. Redox Biol. 2018;14:218–28. 28961512 10.1016/j.redox.2017.09.005 PMC5619994

[31] Zayed MA , Wei X , Park KM , Belaygorod L , Naim U , Harvey J , et al. N‐Acetylcysteine accelerates amputation stump healing in the setting of diabetes. FASEB J. 2017;31:2686–95. 28280002 10.1096/fj.201601348R PMC5434655

[32] Kasperczyk S , Dobrakowski M , Kasperczyk A , Ostalowska A , Birkner E . The administration of N‐acetylcysteine reduces oxidative stress and regulates glutathione metabolism in the blood cells of workers exposed to lead. Clin Toxicol (Phila). 2013;51:480–6. 23731375 10.3109/15563650.2013.802797

[33] Yuan Y , Hilliard G , Ferguson T , Millhorn DE . Cobalt inhibits the interaction between hypoxia‐inducible factor‐alpha and von Hippel‐Lindau protein by direct binding to hypoxia‐inducible factor‐alpha. J Biol Chem. 2003;278:15911–6. 12606543 10.1074/jbc.M300463200

[34] Nguyen QD , Shah SM , Van Anden E , Sung JU , Vitale S , Campochiaro PA . Supplementary oxygen improves diabetic macular edema: a pilot study. Invest Ophthalmol Vis Sci. 2004;45:617–24. 14744906 10.1167/iovs.03-0557

[35] Choudhuri S , Roy PK , Mitra B , Sen S , Sarkar R , Das M , et al. Hyperlipidemia‐mediated increased advanced lipoxidation end products formation, an important factor associated with decreased erythrocyte glucose‐6‐phosphate dehydrogenase activity in mild nonproliferative diabetic retinopathy. Can J Diabetes. 2017;41:82–9. 27916496 10.1016/j.jcjd.2016.07.007

[36] Chettimada S , Gupte R , Rawat D , Gebb SA , McMurtry IF , Gupte SA . Hypoxia‐induced glucose‐6‐phosphate dehydrogenase overexpression and ‐activation in pulmonary artery smooth muscle cells: implication in pulmonary hypertension. Am J Physiol Lung Cell Mol Physiol. 2015;308:L287–300. 25480333 10.1152/ajplung.00229.2014 PMC4338932

[37] Gao L , Mejias R , Echevarria M , Lopez‐Barneo J . Induction of the glucose‐6‐phosphate dehydrogenase gene expression by chronic hypoxia in PC12 cells. FEBS Lett. 2004;569:256–60. 15225644 10.1016/j.febslet.2004.06.004

[38] Larsen BT , Gutterman DD . Hypoxia, coronary dilation, and the pentose phosphate pathway. Am J Physiol Heart Circ Physiol. 2006;290:H2169–71. 16687606 10.1152/ajpheart.00161.2006

[39] Zecchin A , Kalucka J , Dubois C , Carmeliet P . How endothelial cells adapt their metabolism to form vessels in tumors. Front Immunol. 2017;8:1750. 29321777 10.3389/fimmu.2017.01750 PMC5732229

[40] Eelen G , de Zeeuw P , Simons M , Carmeliet P . Endothelial cell metabolism in normal and diseased vasculature. Circ Res. 2015;116:1231–44. 25814684 10.1161/CIRCRESAHA.116.302855 PMC4380230

[41] Koziel A , Jarmuszkiewicz W . Hypoxia and aerobic metabolism adaptations of human endothelial cells. Pflugers Arch. 2017;469:815–27. 28176017 10.1007/s00424-017-1935-9 PMC5438427

[42] Kathagen‐Buhmann A , Schulte A , Weller J , Holz M , Herold‐Mende C , Glass R , et al. Glycolysis and the pentose phosphate pathway are differentially associated with the dichotomous regulation of glioblastoma cell migration versus proliferation. Neuro Oncol. 2016;18:1219–29. 26917237 10.1093/neuonc/now024 PMC4998991

[43] Prescott LF , Park J , Ballantyne A , Adriaenssens P , Proudfoot AT . Treatment of paracetamol (acetaminophen) poisoning with N‐acetylcysteine. Lancet. 1977;2:432–4. 70646 10.1016/s0140-6736(77)90612-2

[44] Dahlin DC , Miwa GT , Lu AY , Nelson SD . N‐acetyl‐p‐benzoquinone imine: a cytochrome P‐450‐mediated oxidation product of acetaminophen. Proc Natl Acad Sci USA. 1984;81:1327–31. 6424115 10.1073/pnas.81.5.1327 PMC344826

[45] Corcoran GB , Todd EL , Racz WJ , Hughes H , Smith CV , Mitchell JR . Effects of N‐acetylcysteine on the disposition and metabolism of acetaminophen in mice. J Pharmacol Exp Ther. 1985;232:857–63. 3973834

[46] Moura FA , de Andrade KQ , dos Santos JC , Araujo OR , Goulart MO . Antioxidant therapy for treatment of inflammatory bowel disease: does it work? Redox Biol. 2015;6:617–39. 26520808 10.1016/j.redox.2015.10.006 PMC4637335

[47] Monsivais SR , Wisdom N , Kruse JL , Davis MC . N‐Acetylcysteine supplementation in an individual with glucose‐6‐phosphate dehydrogenase deficiency‐associated psychosis. Biol Psychiatry. 2016;80:e71–2. 27059873 10.1016/j.biopsych.2016.02.028

[48] Pan S , World CJ , Kovacs CJ , Berk BC . Glucose 6‐phosphate dehydrogenase is regulated through c‐Src‐mediated tyrosine phosphorylation in endothelial cells. Arterioscler Thromb Vasc Biol. 2009;29:895–901. 19359662 10.1161/ATVBAHA.109.184812

[49] Rao X , Duan X , Mao W , Li X , Li Z , Li Q , et al. O‐GlcNAcylation of G6PD promotes the pentose phosphate pathway and tumor growth. Nat Commun. 2015;6:8468. 26399441 10.1038/ncomms9468 PMC4598839

[50] Wang YP , Zhou LS , Zhao YZ , Wang SW , Chen LL , Liu LX , et al. Regulation of G6PD acetylation by SIRT2 and KAT9 modulates NADPH homeostasis and cell survival during oxidative stress. EMBO J. 2014;33:1304–20. 24769394 10.1002/embj.201387224 PMC4194121

[51] Winning S , Splettstoesser F , Fandrey J , Frede S . Acute hypoxia induces HIF‐independent monocyte adhesion to endothelial cells through increased intercellular adhesion molecule‐1 expression: the role of hypoxic inhibition of prolyl hydroxylase activity for the induction of NF‐kappa B. J Immunol. 2010;185:1786–93. 20574001 10.4049/jimmunol.0903244

