# Supplementary material for: N ‐Acetylcysteine protects against cobalt chloride‐induced endothelial dysfunction by enhancing glucose‐6‐phosphate dehydrogenase activity
Source: FEBS Open Bio. 2022 Jun 20;12(8):1475–88. doi: 10.1002/2211-5463.13449 (PMC9340863; doi:10.1002/2211-5463.13449)
Supplement: Supplementary file 1 — Fig. S1. 6‐AN abrogated the protective effect of NAC on arterial endothelial cell damage induced by CoCl2. Fig. S2. CoCl2 treatment to mimic hypoxia effect did not induce ROS accumulation in EA.hy926 cells due to enhanced antioxidative defense system. Fig. S3. G6PD mRNA change upon CoCl2 treatment and CoCl2 dose test. Fig. S4. CoCl2 treatment to mimic hypoxia effect did not induce HAEC apoptosis. [file FEB4-12-1475-s001.pdf]

Supplemental information

Title: N-Acetylcysteine protects against cobalt chloride–induced endothelial dysfunction by enhancing glucose-6-phosphate dehydrogenase activity

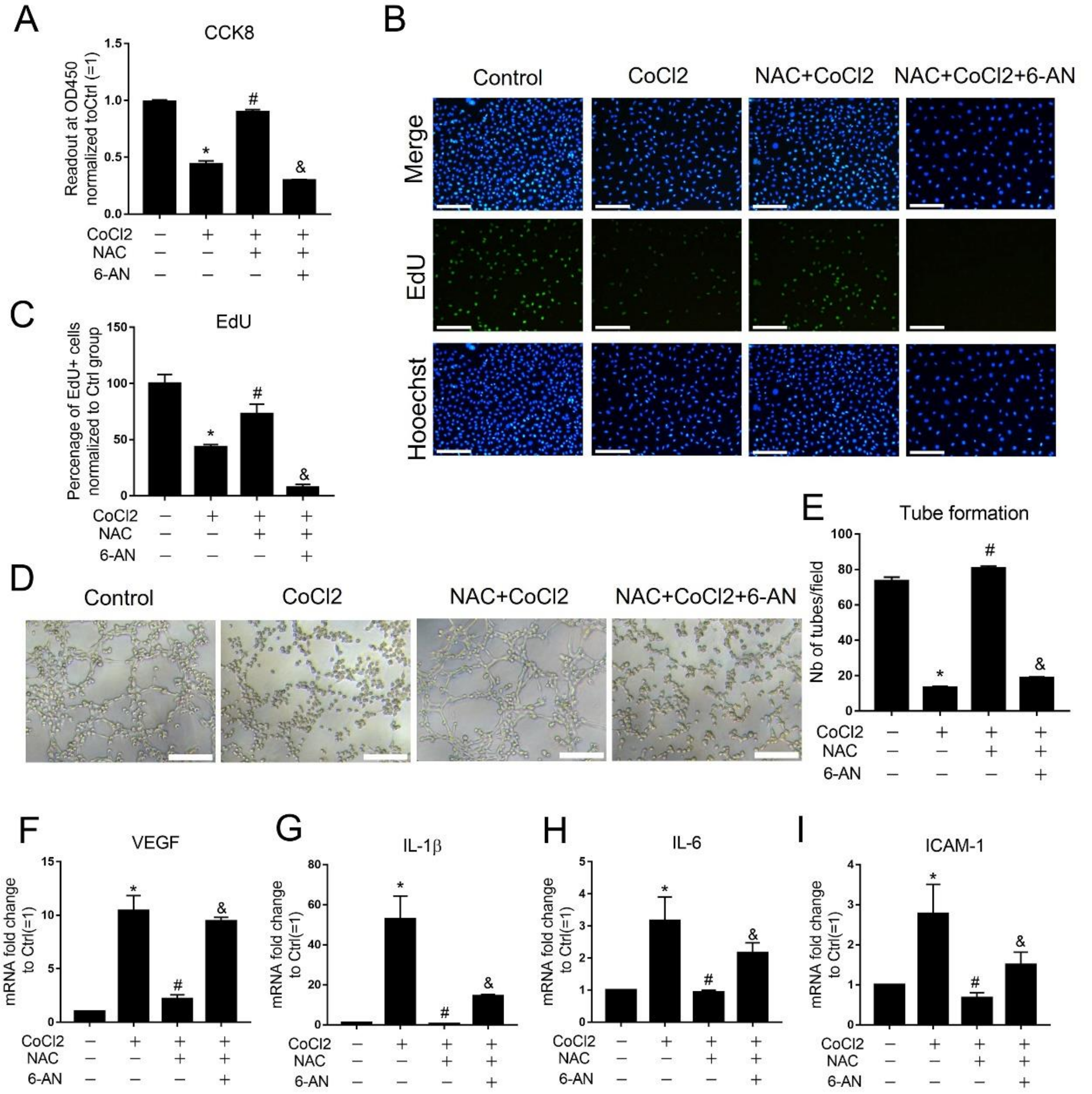

Supplemental Figure 1. 6-AN abrogated the protective effect of NAC on arterial endothelial cell damage induced by $CoCl_2$. Primary human arterial endothelial cells (HAECs) were treated with $CoCl_2$ (400 μM) for 48 h in the presence or absence of NAC (10 mM) or 6-AN (50 μM) pretreatment for 1 hour. Cell viability, proliferation, tube formation capacity and cytokine expression were measured by CCK-8 assay (A), EdU assay (B-C), matrigel assay (D-E) and Real-time PCR (F-I), respectively. Scale bars = 200 μm. Data are expressed as mean ± SEM of independent experiments (n=3). Student's t-test or One-way ANOVA test followed by Tukey's multiple comparison test was used. *P<0.05 vs control group. *P<0.05 vs control group, $^{\#}$P<0.05 vs $CoCl_2$ group, $^{\&}$P<0.05 vs NAC+$CoCl_2$ group.

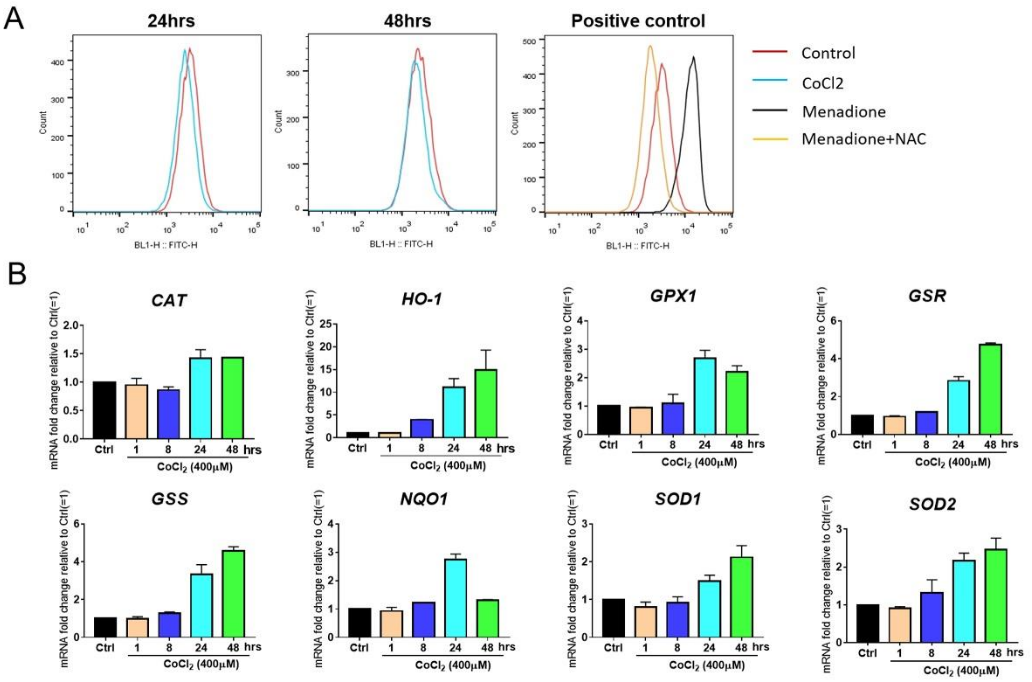

Supplemental Figure 2. $CoCl_2$ treatment to mimic hypoxia effect did not induce ROS accumulation in EA.hy 926 cells due to enhanced antioxidative defense system. (A) Flow cytometry was applied to measure ROS level using Cellrox assay (Thermofisher). As the positive control, Menadione (100 μM) treatment significantly induced ROS formation which was abrogated by NAC (10 mM) pretreatment. However, $CoCl_2$ (400 μM) treatment for 24 or 48 h did not induce ROS formation. Representative flow cytometry histogram of two independent experiments was presented; (B) $CoCl_2$ (400 μM) treatment to mimic hypoxia effect activated antioxidant genes regulated by Nrf2/ARE including *CAT*, *HO-1*, *GPX1*, *GSS*, *GSR*, *NQO1*, *SOD1*, *SOD2*, which mostly peaked up at 24 or 48 h post treatment. Data are expressed as mean ± SEM of independent experiments (n=2).

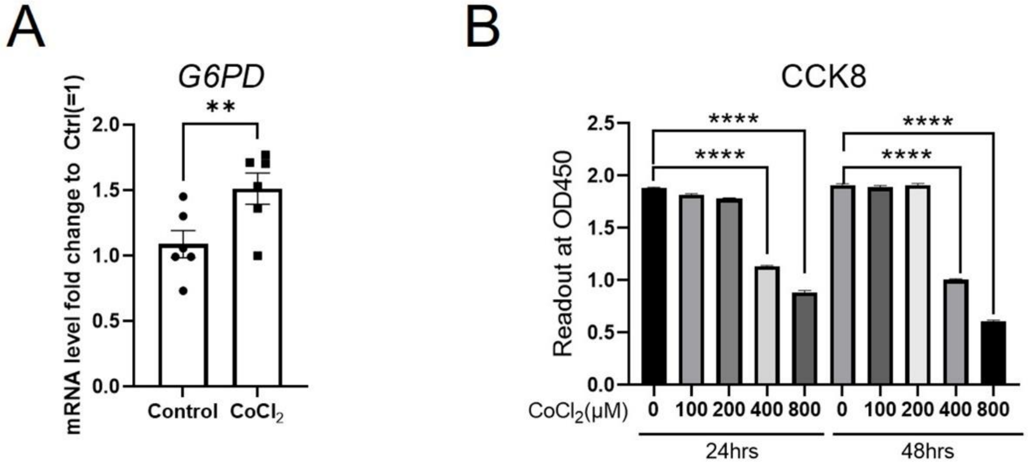

Supplemental Figure 3. G6PD mRNA change upon $CoCl_2$ treatment and $CoCl_2$ dose test. (A) $CoCl_2$ treatment at 400 μM for 48 h increased G6PD mRNA in EA.hy 926 cells (n=6). (B) Treatment with $CoCl_2$ for 24 or 48 h dose-dependently inhibited EA.hy 926 cell viability in CCK8 assay. Data are expressed as mean ± SEM of independent experiments (n=3). Student's t-test or One-way ANOVA test followed by Tukey's multiple comparison test was used. **P<0.01, ****P<0.0001 vs control group.

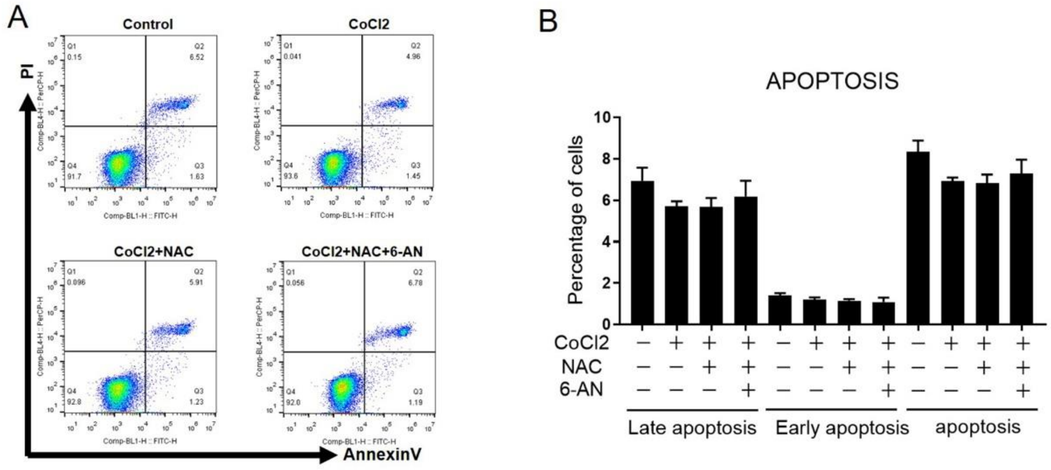

Supplemental Figure 4. $CoCl_2$ treatment to mimic hypoxia effect did not induce HAEC apoptosis. (A) Flow cytometry was applied to measure cell apoptosis in HAECs without (Control group) or with treatment with $CoCl_2$ (400 μM), NAC(10 mM) and 6-AN(50 μM) for 48 h. (B) AnnexinV+/PI- population was defined as early apoptotic cells, AnnexinV+/PI+ was defined as late apoptotic cells. Data are expressed as mean ± SEM of independent experiments (n=4). One-way ANOVA test followed by Tukey's multiple comparison test was used.
